# Drug Repurposing to Fight Colistin and Carbapenem-Resistant Bacteria

**DOI:** 10.3389/fcimb.2019.00193

**Published:** 2019-06-11

**Authors:** Lucie Peyclit, Sophie Alexandra Baron, Jean-Marc Rolain

**Affiliations:** ^1^Faculté de Médecine et de Pharmacie, IRD, APHM, MEPHI, Aix Marseille Univ, Marseille, France; ^2^IHU Méditerranée Infection, Marseille, France

**Keywords:** repurposing, multi-drug resistance (MDR), bacteria, colistin resistance, carbapenam resistant enterobacteriaceae

## Abstract

The emergence of new resistance mechanisms, the failure of classical antibiotics in clinic, the decrease in the development of antibiotics in the industry are all challenges that lead us to consider new strategies for the treatment of infectious diseases. Indeed, in recent years controversy has intensified over strains resistant to carbapenem and/or colistin. Various therapeutic solutions are used to overcome administration of last line antibiotics. In this context, drug repurposing, which consists of using a non-antibiotic compound to treat multi-drug resistant bacteria (MDR), is encouraged. In this review, we first report what may have led to drug repurposing. Main definitions, advantages and drawbacks are summarized. Three major methods are described: phenotypic, computational and serendipity. In a second time we will focus on the current knowledge in drug repurposing for carbapenem and colistin-resistant bacteria with different studies describing repurposed compounds tested on Gram-negative bacteria. Furthermore, we show that drug combination therapies can increase successful by drug repurposing strategy. In conclusion, we discuss the pharmaceutical industries that have little interest in reprofiling drugs due to lack of profits. We also consider what a clinician might think of the indications of these uncommon biologists to treat MDR bacterial infections and avoid therapeutic impasses.

## Introduction

Nowadays, despite recent scientific progress, infectious diseases must always be taken into consideration. The World Health Organization (WHO) closely examines such concerns in order to have an effective health system (World Health Organization, 2018). For 50 years, we have been confronted with the end of the golden age of antibiotic discovery, while some antimicrobial substances have existed for years (Gould, 2016). Due to significant progress that has largely contributed to reducing the number of deaths from infectious diseases, pharmaceutical companies have developed a decreasing interest in these drugs (Conly and Johnston, 2005).

In addition, the use of an antibiotic and the emergence of its resistance are inevitable and intrinsically linked (Mohr, 2016). Although this is not a new phenomenon but a natural one, WHO analysis warns against this serious situation which is the impact, nature and spread of global antimicrobial resistance (Global Antimicrobial Resistance Surveillance System, 2019 Report Early implementation). These resistant bacteria are found in every kind of environment: water, animals, humans, plants and food (Rolain, 2013; Zenati et al., 2016; Bachiri et al., 2017; Tafoukt et al., 2017). The inappropriate use of antimicrobial agents and the spread of antibacterial resistance are among factors that lead to a high rate of resistance in clinical, animals, and even in environmental isolates (Roca et al., 2015; Bassetti et al., 2017). Partly because of drug pressure, resistance can occur more easily and affect all types of antibiotics as for the last-line antibiotics used in human medicine drug for resistant bacterial infections (Biswas et al., 2012). In recent years, we have seen an increase in the use of carbapenems as a result of an increase in the carbapenem resistance of Gram-negative bacteria (GNB) (Diene and Rolain, 2013). For example, Monaco et al. showed in Italy that among 191 clinical strains isolated from November 2013 to April 2014, 178 (93%) *Klebsiella pneumoniae* had KPC enzymes (carbapenemases), with 76 (43%) resistant to colistin (Monaco et al., 2014). Although the same situation has been reported with colistin (Olaitan et al., 2014), it has received more attention: last-line treatments may no longer be effective, increasing the risk of spreading infections (Biswas et al., 2012). To combat frequent epidemics and the challenge of rapid spread, new alternatives to last resort treatments must be considered to avoid treatment failure.

As a result, alternatives to antibiotics to treat resistant germs should be a priority (Bassetti et al., 2017). The use of old drugs can be a solution like “forgotten” antibiotics polymyxins, fosfomycin, minocycline or mecillinam, which are still used in clinical settings (Cassir et al., 2014). There is also a renewed interest in antibiotic combinations to circumvent resistance (Lenhard et al., 2016). For example, the synergistic activity of sulfonamide-associated colistin was evaluated against colistin-resistant clinical bacteria (Okdah et al., 2018). But “non antibiotic” solutions can also been considered as alternatives for the therapeutic management of infections (Aslam et al., 2018). Various studies showed that *Clostridium difficile* can be inhibited using bacteriophages or several ongoing trials use antimicrobial peptides as alternatives or preventive treatments in the future (Aslam et al., 2018).

The fight to treat multi-drug resistant (MDR) infections must also include a change in mentality. Rolain and Baquero denounced the fact that society does not accept the use of toxic but effective antibiotics in treatment of life-threatening infections, but on the other hand society can tolerate potential toxicities of other drugs, such as anti-cancer. With the progress of medicine in the management of adverse reactions and the improved monitoring of antibiotic concentrations, old drugs or dosages rejected due to their adverse effects have to be reconsidered (Rolain and Baquero, 2016). In this way, one other promising alternative on which this review focuses is drug repurposing, also called repositioning (Mercorelli et al., 2018). This therapeutic shift is the subject of several studies in different pathologies including cancer (Sleire et al., 2017), heart diseases (Sun et al., 2018), Alzheimer's disease (Kim, 2015) or depression (Ebada, 2017).

In infectiology, repurposing studies are now being carried out (Torres et al., 2016; Soo et al., 2017; D'Angelo et al., 2018; Zheng et al., 2018; Miró-Canturri et al., 2019). In general, the most common bacteria are first tested or those most at risk or in a therapeutic deadlock. If this review focuses on drug repurposing that have been tested on MDR bacteria, it seems important to precise that resistance is rarely crossed and if a molecule is active on a specific species, this compound will potentially be active regardless of its resistance mechanisms. This is because this molecule affects a new target, generally independent of the antibiotic target, as we will see below with ciclopirox (Carlson-Banning et al., 2013), gallium (Goss et al., 2018), and zidovudine (Elwell et al., 1987). Therefore, it can expand the scope to combinations tested on sensitive GNB as for minocycline and polymyxin B tested with non-antibiotics drugs (Schneider et al., 2017). It offers a diversified and still exploitable field of possibilities (Schneider et al., 2017). For carbapenem and colistin-resistant isolates, a few articles are published on this specificity for which we are striving to synthesize them. The aim is thus to identify an innovative therapeutic strategy against these bacteria in a cost-effective and efficient way.

In this review, we will define drug repurposing and its characteristics. We will then make an inventory of what has already been published as a drug for reuse in general and in particular to address the problem of carbapenem and colistin-resistant bacteria. Finally, we will see what prospects exist for this therapeutic strategy.

## Discovery of an Antibacterial Potential in Non-antibiotic Drugs

### What Does Drug Repurposing Mean?

A drug class is assigned to a molecule to describe and group similary together drugs because of their therapeutic use, their biochemical mechanism, by their way of action or their chemical structure. As defined by Waksman in 1947, “*an antibiotic is a chemical substance, produced by micro-organisms, which has the capacity to inhibit the growth of and even to destroy bacteria and other micro-organisms*” (Mohr, 2016). The current trend therefore seems to be moving away from this definition. Indeed, in recent years, drug repositioning seems to have been “a promising field in drug discovery that identifies new therapeutic opportunities for existing drugs” (Doan et al., 2011). The common idea is that to accelerate discovery of new treatment, using old drugs that could potentially treat disease for which the treatments used no longer work or when we no longer have therapeutic solutions must be used (Langedijk et al., 2015). Sir James Black, pharmacologist and Nobel laureate said in 1888: “*The most fruitful basis for the discovery of a new drug is to start with an old drug*” (Chen et al., 2016). It could help to overcome an initial bottleneck in drug development process. It may therefore be a better compromise between risk and reward than other approaches to drug development (Ashburn and Thor, 2004).

### What Are Advantages and Drawbacks?

Drug repurposing present real economic advantages. All studies about structure, pharmacological properties as bioavailability or safety profiles for example have already been conducted. With these drugs, it is possible to skip preclinical trials because toxicity and pharmacokinetic are already known and a certain hindsight has been taken for several years. Drugs can move directly to Phase 2 to test their effectiveness (Mercorelli et al., 2018).

Repurposing drugs can offer new pathways or targets to study new perspectives for curing diseases. As many antibiotics already affect DNA, membrane or protein translation, other pathways essential for bacterial growth, remain available for activity of molecules, such as assimilation pathways of essential compounds like sugars or amino acids. Combined strategies that reduce resistance can be used to achieve several targets that could affect bacterial metabolism (Mercorelli et al., 2018; Zheng et al., 2018).

Moreover, this method is favorable to academic or small laboratories because of disinterest of pharmaceutical industries. Without patents, these industries do not see any fruitful interest in it because of rapid emerging resistance (Fernandes and Martens, 2017) and a narrower spectrum of activity (Zheng et al., 2018).

On the other hand, this solution cannot be totally miraculous. Drug repurposing does not work all the time due to the high minimal inhibitory concentration (MIC) (Mercorelli et al., 2018) or inconsistent plasma concentrations tolerated in humans. Dose tested for this new indication is important and can lead to human toxicity (Zheng et al., 2018), what society fears (Rolain and Baquero, 2016). Concerning galenic, an optimization of formulation can also be foreseen if a physico-chemical incompatibility is observed.

### How to Process to Repurpose Drugs?

**Figure 1:**
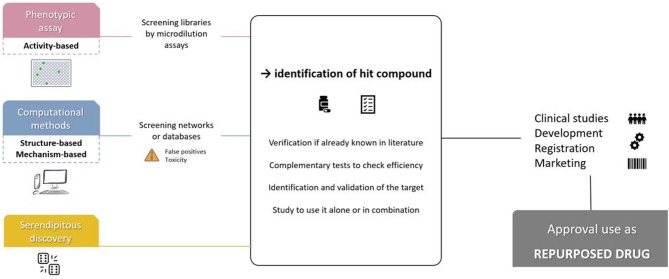
Main steps used for drug repurposing strategy.

Considered an innovative strategy (Doan et al., 2011), three major methods can lead a drug to be repurposed, as shown in Figure 1.

First, phenotypic assay can be performed by high throughput and screening of commercial, public, pharmaceutical compound libraries (Jung et al., 1997; Kim, 2015). These assays consist in classical broth microdilution to identify a hit (Torres et al., 2016). Once antibacterial activity is found, MIC assay is performed to confirm results according to CLSI guidelines (Sun et al., 2016b). Compared to other methods, phenotypic tests have the advantage of being highly physiologically relevant because the effect is observed directly on bacteria (Zheng et al., 2018).

Advances in genomics and bioinformatics modified drug repositioning approach. It consists of *in silico* structure or mechanism-based assays that work with virtual databases. This has been made possible through the prospective development of drug databases and activities, the exchange of information on compounds in collaborative networks and the abundance of resources on the Internet (Hodos et al., 2016). These new calculation tools make it possible to analyse all the different data accumulated in the field that man alone cannot study because they are too complex. This can ensure the understanding and prediction of molecules by generating hypotheses about biological mechanisms (Hodos et al., 2016). Limit to these approaches is that pathways, targets or other data must be already known. Moreover, false positive and toxicity are problematic issues found after identification of a hit (Mercorelli et al., 2018). However, for emerging diseases, this could be a quick way to find an effective molecule as was done for the coronavirus in 2013 (Law et al., 2013).

The last approach is random discovery and can include all types of configurations. Indeed, the side effects of one drug in one disease may be effective for another, such as for the antidepressant bupropion reoriented as an anti-tobacco drug (Hodos et al., 2016). It can be mere coincidence as sulfamides were known for antibacterial properties and finally also employed for antidiabetic ones (Deuil, 1956). However, these unexpected observations could also potentially be identified by informatics methods, in view of knowledge of compound side effects (Hodos et al., 2016).

Despite all these techniques, if a molecule is identified, it must then go through the steps of its clinical evaluation.

## Current Knowledge in Drug Repurposing for Carbapenem and Colistin Resistance?

Studies generally screen MDR bacteria to ensure a broader spectrum of action (Hijazi et al., 2018) and sometimes bacteria only with colistin or carbapenem resistance to treat the ongoing issue (Ayerbe-Algaba et al., 2018). Several major studies have performed drug repurposing on MDR bacteria belonging to the ESKAPE (*Enteroccocus faecium, S. aureus, K. pneumoniae, A. baumannii, Pseudomonas aeruginosa, Enterobacter* species) group (Table 1). Seven non-antibacterial compounds inhibited the growth of an *Acinetobacter baumannii* strain resistant to most antibiotics including carbapenems: 3 antineoplastics (5-fluorouracil, 6-thioguanine and pifithrin-μ), 1 anti-rheumatic (auranofin), 1 antipsychotic (fluspirilene), 1 anti-inflammatory (Bay 11-7082), and 1 alcohol deterrent (disulfiram). Five-fluorouracil and 6-thioguanine seemed to be the best candidates for repurposing to treat MDR clinical *A. baumannii*. Their IC90 values or MIC were lower than standard plasma drug concentration levels in human, suggesting a possible use without major adverse events (Cheng et al., 2019).

**Table 1:** Relevant repurposing reports for carbapenem and colistin resistant bacteria.

	**Compound**	**Approved use or known as**	**Activity -alone or in combination with-**	**Tested bacteria**	**Resistance phenotypes (and/or)**	**References**
Infectiology	Zidovudine	Antiretroviral	Alone	*Escherichia coli* *Klebsiella pneumoniae*	Carbapenems Colistin	Peyclit et al., 2018
			Colistin	*E. coli* *K. pneumoniae* *Enterobacter cloacae* *Pseudomonas* * aeruginosa* *Acinetobacter* * baumannii*	Carbapenems Colistin	Hu et al., 2018; Loose et al., 2018
			Tigecycline	*E. coli* *K. pneumoniae*	Carbapenems	Ng et al., 2018
	Niclosamide	Anthelminthic	Colistin	*A. baumannii* *K. pneumoniae*	Colistin	Ayerbe-Algaba et al., 2018
			Alone			Cebrero-Cangueiro et al., 2018
	Pentamidine	Antiprotozoal	Rifampicin	*K. pneumoniae* *E. coli* *E. cloacae*	Carbapenems Colistin	Cebrero-Cangueiro et al., 2018
						
			Aminoglycosides			Cebrero-Cangueiro et al., 2018
	Ciclopirox	Antifungal	Alone	*E. coli* *K. pneumoniae* *A. baumannii*	Carbapenems	Carlson-Banning et al., 2013
Oncology	5-fluorouracil	Antineoplastic	Zidovudine	*A. baumannii*	Carbapenems	Cheng et al., 2019
	Mitotane	Antineoplastic	Polymyxin B	*A. baumannii* *P. aeruginosa* *K. pneumoniae*	Carbapenems Polymyxin	Tran et al., 2018
	Gallium	Antineoplastic	Alone	ESKAPE species	MDR	Hijazi et al., 2018
	Tamoxifen Raloxifen Toremifen	SERM	Polymyxin B	*P. aeruginosa* *K. pneumoniae* *A. baumannii*	Colistin	Hussein et al., 2017
Central Nervous System	Sertraline	Antidepressant	Polymyxin B	*P. aeruginosa* *K. pneumoniae*	Colistin Carbapenems	Otto et al., 2019
	Citalopram	Antidepressant	Polymyxin B	*A. baumannii* *E. coli* *K. pneumoniae*	Colistin Carbapenems	Otto et al., 2019
	Fluspirilene	Antipsychotic	Colistin	*A. baumannii*	Carbapenems	Cheng et al., 2019
Metabolism	Bay 11-7082	Anti-inflammatory	Colistin	*A. baumannii*	Carbapenems	Cheng et al., 2019
	Spironolactone	Diuretic	Polymyxin B	*E. coli*	Carbapenems	Otto et al., 2019
Natural compound	Resveratrol	Stilbene	Alone	*E. coli* *Enterobacter* * aerogenes*		Seukep et al., 2016
			Streptomycin	*K. pneumoniae*	MDR	Seukep et al., 2016
			Ciprofloxacin	*P. aeruginosa* * Providencia stuartii* *E. cloacae*		Seukep et al., 2016
			Colistin	*E. coli* *K. pneumoniae* *A. baumannii* *Serratia marcescens* *Proteus mirabilis*	Colistin Carbapenems	Cannatelli et al., 2018
	Pterostilbene	Anticancer Antioxidant	Polymyxin B	*K. pneumoniae*	Colistin	Zhou et al., 2018
	Eugenol	Essential oil	Colistin	*E. coli*	Colistin	Wang et al., 2018

*SERM, Selective estrogen receptor modulator*.

**Figure 2:**
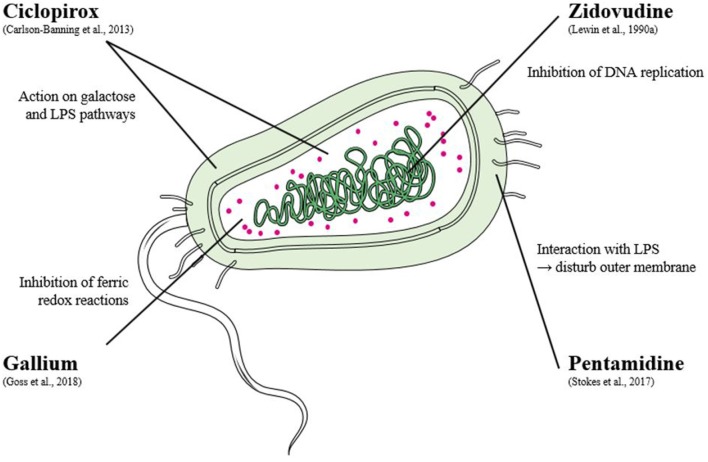
Mechanisms of action of compounds tested alone on colistin or carbapenem resistant bacteria.

All mechanisms of action and targets are considered because the objective is to escape therapeutic drug classes. Each repurposed molecule can be used to study a new pathway (Figure 2). An antifungal agent developed nearly forty years ago, ciclopirox, also has good repurposing criteria, as shown by an American study conducted in 2013 (Carlson-Banning et al., 2013). Due to its excellent safety profile, it has already been repurposed in various pathologies such as myeloma, or as an anti-human immunodeficiency virus drug. It prevents enzyme actions, essential for cellular metabolism or functions, by inhibiting the availability of co-factors. Its activity was proved against MDR *E. coli, K. pneumoniae*, and *A. baumannii* strains. They demonstrated a novel mechanism of action: ciclopirox affects the galactose and LPS salvage pathways (Carlson-Banning et al., 2013). In addition, the bacterial activity of gallium has been known for more than 80 years but is first used as an anti-cancer agent. Due to its chemical similarity to iron, gallium inhibits ferric redox reactions or pathways, and then bacterial growth. In this matter, it has a broad spectrum of activity, in particular MDR ESKAPE pathogens (Rangel-Vega et al., 2015; Hijazi et al., 2018). In fact, a phase 2 trial in cystic fibrosis patients assess the activity of gallium and suggests its safety and efficacy for human infections (Goss et al., 2018). This once again represents potential and promising targets for the control of infectious germs.

It can be noticed that all pharmaceutical classes can be involved, from anticancer to anti-inflammatory and also antiparasitic drugs. Pachón-Ibáñez et al. and the study of Stokes (Stokes et al., 2017) of the previous year showed that pentamidine was effective against polymyxin resistant strains. This antiprotozoal agent usually effective in trypanosomiasis, leishmaniasis and some fungal infections was here tested against 8 *Enterobacteriaceae* (5 *K. pneumoniae*, 1 *E. coli* and 2 *Enterobacter cloacae*). Pentamidine was bactericidal for 7 strains which carried out carbapenemases or showed colistin resistance. Moreover, these effects potentiated activity of other antibiotics due to a synergistic activity with rifampicin, or aminoglycosides for *E. cloacae*. With rifampicin, combination was effective against most of the strains tested (Cebrero-Cangueiro et al., 2018).

In this context of drug reprofiling, various studies identified the antiretroviral zidovudine, also called azidothymidine (AZT), as an active molecule against resistant *Enterobacteriaceae* (Doléans-Jordheim et al., 2011; Peyclit et al., 2018). The interest in drug repurposing in MDR enterobacterial infections has revived the forgotten antibacterial properties of this drug mentioned for the first time in 1986 (Elwell et al., 1987). On a series of *Enterobacteriaceae* with different colistin resistance profiles (*mcr-1* gene, *mgrB* or *pmrB* mutations), its antibacterial action was confirmed with MICs ranging from 0.2 and 6.25 μM. Pharmacokinetic data showed that AZT concentrations found would be compatible with plasma concentrations obtained for doses used in human medicine (Peyclit et al., 2018). Due to a relatively rapid mutation frequency (Doléans-Jordheim et al., 2011) and resistant strains already reported (Lewin et al., 1990a), it would appear that zidovudine is more suitable for use in combination. Indeed, zidovudine was tested in various associations with antibiotics from different class (Lewin et al., 1990b; Mascellino et al., 1993). In a recent article (Hu et al., 2018), checkerboard analysis with colistin showed synergistic activity against 60.87% of the Extended spectrum ß-lactamases (ESBL) *E. coli*, 87.1% of the ESBL *K. pneumoniae*, 100% of NDM-1 producing strains and 92.31% of colistin resistant (*mcr*-1) *E. coli*. With this bactericidal combination, the activity of colistin has been improved, which could reduce the dose of colistin for a better effect (Hu et al., 2018). Patented in 2014 (Hu and Coates, 2014), it has been enrolled in a Phase 1 clinical trial. Results showed that the association had a bactericidal activity on plasma concentration on *mcr-1* positive strains and that it was well tolerated by the healthy volunteers involved in the study (Loose et al., 2018). Further human studies can be undertaken to confirm these results, but they confirm that AZT can be a recovery therapy against MDR bacteria and thus help clinicians avoid therapeutic impasses.

Finally, in order to anticipate the emergence of resistance in bacteria, some molecules have the significant advantage of not being used alone, of focusing on multiple targets and thus eradicating the infection as quickly as possible.

### Drug Combination Therapy Increases Successful Drug Repurposing

After finding a positive response to a new use, if it does not sufficiently meet the criteria of efficacy, safety, pharmacodynamics, non-toxicity, combination studies with another drug may be considered in order to use it effectively. Drug combinations consist on an association of two or more drugs in order to enhance efficacy of therapeutic strategy and increase chances of clinical applications. It broadens the spectrum of activity of useful antibiotics, for example for serious infections requiring urgent and effective treatment (Zheng et al., 2018). The use of two or more drugs has an impact on different targets, increasing the impairment of microbial function and reducing the risk of resistance emergence (Zheng et al., 2018). The main goal of drug association is to produce a synergistic effect: effect produced by combination is greater than that achieved with any of the drugs used alone. Moreover, if one compound has low activity, another can potentiate and increase it. This reduces the concentration of each individual molecule and can therefore be used at lower doses. This is a real advantage when one knows toxicity of certain drugs (Sun et al., 2016a; Zheng et al., 2018). Sun et al. showed the use of drug combinations reduced toxicity. It increased activity compared to a single therapy when cytotoxicity was proven allowing the use of some drugs in human medicine that were not conceivable on their own (Sun et al., 2016a).

### Drug Repurposing for Combination With Known Antibiotics

Research in drug repurposing for combination with known antibiotics on carbapenem and/or colistin-resistant bacteria has mainly been conducted in association with polymyxins drugs (Table 1). Niclosamide, an anthelmintic drug, known to be active against most tapeworms, seems to interact with the negatively charged outer membrane of colistin-resistant strains leading to a synergistic effect with colistin. This effect was observed on 18 strains with 13 colistin-resistant *A. baumannii* (*pmrB* altered), and 2 colistin-resistant *K. pneumoniae* (*mgrB* and *pmrB* altered) (Ayerbe-Algaba et al., 2018). Colistin combination therapy with selective estrogen receptor modulators (SERM) as tamoxifen, raloxifen and toremifen also exhibited good activity against polymyxin-resistant *P. aeruginosa, K. pneumoniae*, and *A. baumannii*. Tested *in-vitro* concentrations could be achievable for human concentration (Hussein et al., 2017; Schneider et al., 2017). In 2019, fluspirilene and Bay 11-7082 have shown promising results by resensitizing a resistant *A. baumannii* to overcome colistin resistance (Cheng et al., 2019). Regarding polymyxin B, synergistic activity with mitotane, an antineoplastic approved for carcinoma treatment, was studied *in-vitro* on 10 strains including carbapenem or polymyxin-resistant GNB. Tests were also carried out on infections of mouse burn wounds, which led to a promising result for the treatment of this type of infection (Tran et al., 2018). Using knowledge of the mechanisms of action, an approach was also tested with some channel-blocking molecules. Indeed, these efflux pump inhibitors did not have an effect on bacteria alone but combined with an antibiotic, they demonstrated restoration of its activity. The effect is more or less strong but as for neuroleptics (prochlorperazine, chlorpromazine, promazine) associated with meropenem appear to be effective against MDR *A. baumannii* (Yang and Chua, 2013).

Otto et al., showed a potential efficacy of 7 drugs, including 3 antidepressants (amitriptyline, imipramine and sertraline) and 4 antipsychotics (chlorpromazine, clonazepam, haloperidol, and levopromazine) with polymyxin B against 20 tested GNB displaying various resistance mechanisms including carbapenemases (Otto et al., 2019). Only sertraline, chlorpromazine and levopromazine had a synergism effect with polymyxin B against *A. baumannii, E. coli* and *K. pneumoniae* isolates. Among all non-antibiotics, only spironolactone, which had only a good efficacy against *E. coli* isolates, showed non-toxic levels of minimum concentration for synergy with polymyxin B (Otto et al., 2019). These findings show that non-antibiotics molecules can be effective in combination but studies need to be pursued to develop association with effective concentrations that are clinically tolerated (Otto et al., 2019).

### Natural Compounds for New Combinations Should Not Be Excluded

Although some natural compounds are not FDA-approved, some may also be part of this process for which a compound is used in another known property. The stilbene and polyphenol resveratrol is produced by various plants (as grapes and blueberries) and is known to have various antioxidant properties and chemopreventive activities. In the bark from *Nauclea pobeguinii*, Cameroonian researchers found this compound and tested it on GNB with MDR phenotypes. It was active alone and in a synergy with streptomycin and ciprofloxacin (Seukep et al., 2016). Rossolini et al. demonstrated in 2018 activity of resveratrol as an antimicrobial agent in combination with colistin on a panel of colistin-resistant (chromosomic or plasmid resistance) GNB (Cannatelli et al., 2018). Resveratrol seems to potentiate colistin activity and thus makes it possible to restore its action among different species and resistance pathways. Thus, another natural compound, pterostilbene, derived from blueberries and grapes and known for its anticancer, anti-inflammatory and antioxidant effects, appears to enhance polymyxin activity *in vitro* and *in vivo* (Zhou et al., 2018). They demonstrated synergistic effects with colistin and with polymyxin B in *mcr-1* positive strains by 8-fold reducing MIC of polymyxin B with 32μg/ml (Zhou et al., 2018). Pterostilbene could therefore affect *mcr-1* function and restore antibacterial activity of polymyxin B in resistant isolates. Probably safe for human clinical practice and possibly not exerting selective pressure associated with the current ATB, it is therefore considered a good candidate for drug repurposing (Zhou et al., 2018). Lastly, eugenol is a phenylpropanoid present in essential oil of many plants. Thirteen animal *E. coli* strains with colistin resistance were subjected to MIC, time kill and checkerboard assays to evaluate combination between colistin and eugenol (Wang et al., 2018). They observed that *mcr-1* gene expression was down regulated by eugenol and suggested a possible binding between eugenol and MCR-1 protein. (Wang et al., 2018).

**Figure 3:**
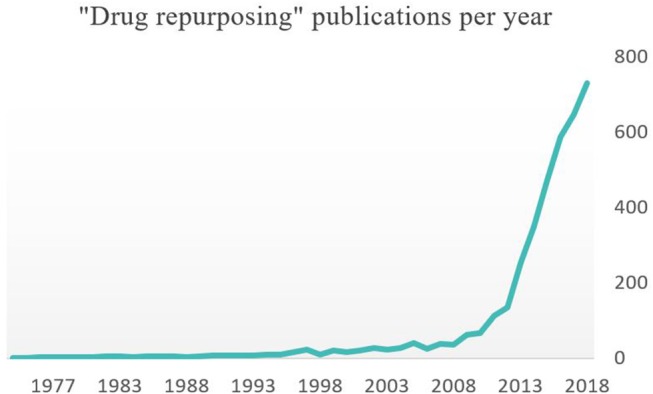
Annual number of publications on PubMed search engine with “drug repurposing” keyword.

All these molecules are still being tested *in vitro* or in clinical trials and none have yet received new indication for MDR infection treatment. However, research on drug repurposing is gaining new dynamism if we can refer to the number of annual publications in recent years (Figure 3).

## Perspectives

In conclusion, this review therefore addresses two main aspects, both the emerging drug repurposing strategy and resistance to last-line antibiotics, carbapenems, and colistin. A new economic model is to be considered for antibiotic development because industries do not seem interested in this new strategy (Zheng et al., 2018). Indeed, as antibiotics are not part of chronic treatment strategies, this could not be as economically attractive (Conly and Johnston, 2005). This disinterest in antibiotics research is reflected in their absence in programs of future developments of major pharmaceutical companies (Spellberg et al., 2004). Start-ups or small companies, on the other hand, can see an interest in taking back antibiotics that have failed in clinical phases, for example. They believe they have different drug development strategies and do not require as many benefits to cover their costs compared to multinational pharmaceutical companies (Fernandes and Martens, 2017). However, Phase 3 clinical trials for new and repurposed drugs remain very expensive: it is estimated between $40 million and $300 million of USD (Azvolinsky, 2017). With such a budget, this does not work in favor of small firms.

As for the question of how to treat bacterial resistance, only one answer has not been found and the future offers us new possibilities. Various strategies are being considered as treatment using fecal microbiota (Davido et al., 2017), antimicrobial peptides (Hashemi et al., 2017) or bacteriophages (Parmar et al., 2017). A *Streptomyces sp*. present in alkaline soil in Ireland has been in the spotlight recently to inhibit growth of MDR bacteria (Terra et al., 2018) which reminds of Flemming discovery. Additionally, the discovery and studies on the CRISPR/Case9 system may suggest that it may be the ultimate weapon to fight infectious diseases and thus control antibiotic resistance (Doerflinger et al., 2017).

**Figure 4:**
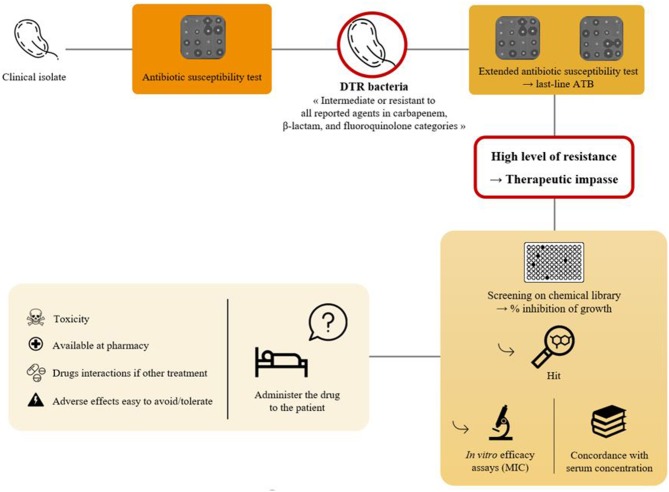
How to proceed with a DTR (difficult-to-treat resistant) bacteria.

Question of drug repurposing remains rather wide. Although this seems to be a better solution, drug combinations can also lead to adverse interactions. First, toxic side effects can be increased. Then, concerning compound galenic, physico-chemical interactions and differences in stability, solubility and conservation can result from the combination of two molecules making it incompatible. Formulation then becomes more complicated (Sun et al., 2016a). On the other hand, we must change this vision where each drug belongs to only one box. Clinicians may have difficulty understanding why a biologist recommends the use of an anti-cancer or anti-inflammatory drug to treat their cystic fibrosis patient's bacteremia rather than a last resort antibiotic they have always used. Communication in this sense remains essential between health professionals and clinical studies to prove these activities are critical. However, with current knowledge on drug repurposing as antibacterial agents and the problematic to find an alternative therapeutic in some situations, screening of non-antibiotics in a “à la carte” way can be an issue (Figure 4). In the area of personalized medicine, we could imagine a personalized antibiotic susceptibility testing in case of infection caused by a highly resistant bacterium. If all last-line antibiotics have been tested and appear insufficient to successfully treat the patient, testing non-antibiotic drugs that are potentially active on the pathogen, alone or in combination with antibiotics, could help clinicians use these drugs. As previously reported by Kadri et al., these difficult-to-threat bacteria refer to bacteria that are resistant to all first-line antibiotics. It represents less than 1% of isolates and those that are resistant to second-line antibiotics are even rare (Kadri et al., 2018). This solution, combined with monitoring of serum levels and adverse events such as dialysis of a nephrotoxic drug (Rolain and Baquero, 2016), could offer great potential for treating a patient with this MDR bacterium. The problem remains to be able to routinely test a large panel of molecules, in an automated, reproducible, and not too expensive way.

## Author Contributions

LP, SB, and J-MR drafted and revised the manuscript. All authors read and approved the final manuscript.

## Conflict of Interest Statement

The authors declare that the research was conducted in the absence of any commercial or financial relationships that could be construed as a potential conflict of interest.
